# Fracture of the Skull with Protrusion of Brain Substance and Removal of Same

**Published:** 1893-08

**Authors:** W. R. Googe

**Affiliations:** Abbeville, Ga.


### FRACTURE OF THE SKULL WITH PROTRUSION OF BRAIN SUBSTANCE AND REMOVAL OF SAME.

BY W. R. GOOGE, M. D., ABBEVILLE, GA.

It is only recently that intrusion on the brain and membranes has been attended by so small a mortality ; but owing to the great success and progress which has been achieved in antiseptic surgery, it is now of common occurence.

It is not the purpose of this paper to let new light into the subject of brain surgery, but simply to corroborate the fact that if thorough cleanliness and strict antiseptic precaution are strenuously observed, that the time will come, and to a certain extent now is, that the surgeon may penetrate the most secluded recesses of the human mechanism, and perform wonders which were not long ago shrouded in obscurity. .

The time has been, and not long ago the idea was prevalent that intrusion on the brain or membranes was attended by almost certain death ; or to penetrate the abdominal walls and peritoneum was considered folly of the most aggravated type, but thanks to the promulgators of antisepsis, we can now, with impunity, perform operations of this nature with a very low per cent. of mortality, which none dare to dispute. It is a boon to humanity and should be a source of self-congratulation on the part of every physician.

Fracture of the cranium with protrusion of brain substance and removal of same, with recovery, is now regarded among the laity and also among some of the practitioners of medicine as an impossibility, and it is the intention of the writer to disclose the results of a case of this description, and to show, if any doubt exists, that recovery in such instances is of very common occurrence.

On December 18th, of last year, Mr. A. B. Pemberton, while guarding convicts for the Ocmulgee Brick Company, at Abbeville, Ga., was struck with the eye of a common club axe on the posterior portion of the skull, near the articulation of the occipital and parietal bones, which resulted in a complete fracture, with the bone depressed and resting on the brain substance. The depressed bone had loosened quite a quantity of the brain, which, after observing the usual antiseptic precautions, I very carefully removed. It weighed just a little over half an ounce.

After carefully removing every particle of matter that could possibly be foreign, with a common elevator I raised the skull to its proper position and placed it in apposition, after which I dressed the wound with iodoform gauze, packing the wound in order to prevent the healing process of it externally, after which he was carried home and put to bed. Strange to say, he could walk and talk sensibly, still he had no recollection of the matter afterwards.

December 19. Temperature 100° F. Absence of sensation, not being able to feel the prick of a pin. Mind wandering. Gave sulphate of quinine, and ordered warm soapsuds and water every day.

December 20. Temperature 101° F. Slight sensation. Able to talk, but would lose the subject.

December 21. Temperature 100° F. Sensation returning and complaining of pain in left arm. Wound was on right side of head.

December 22. Temperature 99½° F. Pain more severe on left side. Sensation almost restored. Gave hypodermic of morphia and atropine.

December 23. Pain gone. Sensation restored. Appetite good. Had eaten nothing since injured, being very much nauseated all the time.

December 25. Temperature normal. Dressed wound and strange to say found no pus whatever. Patient able to sit up for dressing wound.

December 27. Patient walked up town, a distance of one quarter of a mile, contrary to my directions.

January 1. Bone had healed, and removed packing from wound.

January 5. Wound granulating nicely.

January 10. Discharged patient with the wound almost well.

January 28. Patient thoroughly well and accepted a position on S. F. & W. R. R., as section master.

The question might naturally arise, are you sure that you removed the brain substance, and in reply I would say that I was never surer of anything in my life, and can corroborate every assertion by my associate, Dr. A. R. Royal.

---

CURETTING THE LACRYMAL SAC WITHOUT CUTANEOUS INCISION.—Terson (*Ann. d'oculistique*, April, 1892,) advises the following method of operation: After injecting a few drops of a solution of cocaine into the lacrymal sac, the upper canaliculus is freely divided, and the entire passage down to the end of the nasal duct is opened by a Bownan's probe No. 4. Then a small cutting fenestrated curette is pushed into the lacrymonasal canal up to the handle. The curette is then drawn upward, cutting sharply through the lining membrane, and again pushed down and draw up, always revolving the handle each time until the entire inner surface of the canal has been thoroughly scraped.—*Ex.*

